# Supplementary material for: Chromothripsis during telomere crisis is independent of NHEJ, and consistent with a replicative origin
Source: Genome Res. 2019 May;29(5):737–49. doi: 10.1101/gr.240705.118 (PMC6499312; doi:10.1101/gr.240705.118)
Supplement: Supplemental Material [file supp_gr.240705.118_Supplemental_file_1.zip › contigs/annotated_contigs/DB48/contig.2.DB48_length_530_mean_cov_4.24528301887.docx]

**DB48_length_530_mean_cov_4.24528301887**

```
TGATCCTGGCTTTGTAAATATTCCCTTTTCCCAACAAAGTGTATTCCTTAATCCATGCCATATGTCATTGTGGAATTTCTCTGGATCTC
 >chr11:108690751-108691103 - E=3e-200
TGATGACAAGTGCCAATCCAGATGGGACCTACCTGACAAATCTAAACCTTTCAGAATTTAGTGAAGAGTAGTTAATAACAGATGCCATG

TGCCAAACACCACGCTAGATGTTTTATATTTCATTTACTCGGCACAACCACCCTTTTGAGGAACTGCCTGCATTTTACAGATGATGAAA

TAATTATTCAGCTAAAAAGGACAGAAGCATGCTCTAAACTCAGGTTTATCAAACATCAAAATTTATCTTCTTGAGCTTTGAAGCT|CAA

CACTTCTATTCAACACAGTACTAGAAGTCTTAGCCAGAGCAATGAGACAAGAGAAAGAAATAAAGGGAAACCAAATCAGTAAAGAGTAA
 >chr11:108701148-108701323 - E=1e-93
ATCAAACTGTCGCTGTTTGCTGATGATATGATTGTATAACTAGAAAACACTAAAGACTCATCCAAAAAGCTCCCAGAACCGGTAAA
```
